# An Unsupervised Data-Driven Anomaly Detection Approach for Adverse Health Conditions in People Living With Dementia: Cohort Study

**DOI:** 10.2196/38211

**Published:** 2022-09-19

**Authors:** Nivedita Bijlani, Ramin Nilforooshan, Samaneh Kouchaki

**Affiliations:** 1 Centre for Vision, Speech and Signal Processing University of Surrey Guildford United Kingdom; 2 Surrey and Borders Partnership NHS Foundation Trust Guildford United Kingdom; 3 Care Research and Technology Centre UK Dementia Research Institute Imperial College London United Kingdom; 4 School of Psychology University of Surrey Guildford United Kingdom

**Keywords:** contextual matrix profile, multidimensional anomaly detection, outlier detection, sensor-based remote health monitoring, dementia, unsupervised learning

## Abstract

**Background:**

Sensor-based remote health monitoring can be used for the timely detection of health deterioration in people living with dementia with minimal impact on their day-to-day living. Anomaly detection approaches have been widely applied in various domains, including remote health monitoring. However, current approaches are challenged by noisy, multivariate data and low generalizability.

**Objective:**

This study aims to develop an online, lightweight unsupervised learning–based approach to detect anomalies representing adverse health conditions using activity changes in people living with dementia. We demonstrated its effectiveness over state-of-the-art methods on a real-world data set of 9363 days collected from 15 participant households by the UK Dementia Research Institute between August 2019 and July 2021. Our approach was applied to household movement data to detect urinary tract infections (UTIs) and hospitalizations.

**Methods:**

We propose and evaluate a solution based on Contextual Matrix Profile (CMP), an exact, ultrafast distance-based anomaly detection algorithm. Using daily aggregated household movement data collected via passive infrared sensors, we generated CMPs for location-wise sensor counts, duration, and change in hourly movement patterns for each patient. We computed a normalized anomaly score in 2 ways: by combining univariate CMPs and by developing a multidimensional CMP. The performance of our method was evaluated relative to Angle-Based Outlier Detection, Copula-Based Outlier Detection, and Lightweight Online Detector of Anomalies. We used the multidimensional CMP to discover and present the important features associated with adverse health conditions in people living with dementia.

**Results:**

The multidimensional CMP yielded, on average, 84.3% recall with 32.1 alerts, or a 5.1% alert rate, offering the best balance of recall and relative precision compared with Copula-Based and Angle-Based Outlier Detection and Lightweight Online Detector of Anomalies when evaluated for UTI and hospitalization. Midnight to 6 AM bathroom activity was shown to be the most important cross-patient digital biomarker of anomalies indicative of UTI, contributing approximately 30% to the anomaly score. We also demonstrated how CMP-based anomaly scoring can be used for a cross-patient view of anomaly patterns.

**Conclusions:**

To the best of our knowledge, this is the first real-world study to adapt the CMP to continuous anomaly detection in a health care scenario. The CMP inherits the speed, accuracy, and simplicity of the Matrix Profile, providing configurability, the ability to denoise and detect patterns, and explainability to clinical practitioners. We addressed the need for anomaly scoring in multivariate time series health care data by developing the multidimensional CMP. With high sensitivity, a low alert rate, better overall performance than state-of-the-art methods, and the ability to discover digital biomarkers of anomalies, the CMP is a clinically meaningful unsupervised anomaly detection technique extensible to multimodal data for dementia and other health care scenarios.

## Introduction

### Background

Dementia is a progressive and irreversible decline in a wide range of brain activities, including impaired memory, thinking, orientation, comprehension, calculation, learning capacity, language, and judgment, beyond what might be expected from natural biological aging. The World Health Organization estimates that approximately 55 million people have dementia worldwide, which is set to rise to 78 million in 2030 and 139 million in 2050 [1]. Managing the care of this growing population incurs significant costs. The Alzheimer’s Society puts the cost of care for people with dementia in the United Kingdom at GBP 34.7 billion (US $40 billion), rising sharply to GBP 94.1 billion (US $108.6 billion) by 2040 [2]. The hospitalization of people living with dementia because of potentially preventable conditions such as fall injuries, sepsis, pneumonia, and urinary tract infection (UTI) puts huge pressure on health systems. To minimize preventable hospitalizations, there is a significant investment in artificial intelligence–driven technologies that enable the health of people living with dementia to be remotely monitored and assisted while they live in the comfort of their own homes.

The UK Dementia Research Institute Care Research and Technology Centre has made a significant effort in this direction with its vision to “use patient-centered technology to help people affected by dementia to live better and longer in their own homes” [3]. The team at the UK Dementia Research Institute Care Research and Technology Centre has developed a sensor-based remote health monitoring platform that enables clinicians to intervene early and allows researchers to improve their understanding of dementia onset and progression [4]. The cohort currently covers 102 people with dementia living with their caregivers in their own homes. Data collection commenced in 2019 and will continue until at least 2025, with more participants being onboarded each year, making it one of the largest, longest-running, and most diverse and unique dementia data collection programs worldwide. The sensors, framework, models, clinical monitoring workflows, app for participants, and monitoring dashboard together form a digital platform called Minder (please see the website of the UK Dementia Research Institute [4] for more information).

Occasionally, people with dementia present with behavioral and psychological symptoms such as agitation, aggression, sleep disturbances, urinary system disorders, dehydration, and falls. UTI is the most diagnosed infection in older adults, and early identification is key to preventing further complications [5,6]. The diagnosis of UTI remains problematic because of the presence of a range of nonspecific symptoms, a high prevalence of asymptomatic bacteriuria, and reduced help-seeking behavior [7-9].

An “anomaly” in the context of home health monitoring can be simply understood as an unexpected but significant irregularity in otherwise normal data, which is indicative of an adverse condition. Anomalies are difficult to detect within overwhelming volumes of normal data. The cost of missing or misclassifying anomalies can be high (eg, failing to detect a UTI could be catastrophic). Current methods for health care anomaly detection are challenged by one or more real-world issues: high-dimensional and multivariate data; little to no information on the distinction between normal and abnormal data; time course data and the need to make predictions with low latency; patient-to-patient variability; noise and lack of periodicity because of social visits, pets, sensor issues, and noisy labels; high false alert rate; high tuning needs; and low explainability to clinical monitoring teams and caregivers [10].

The aim of our work was to develop a clinically useful, domain-agnostic, fast, lightweight, unsupervised anomaly detection approach for real-world noisy health care data. We accounted for individual variability, generalizability across individuals and domains, and explainability to clinicians and carers in the form of digital biomarker discovery. Our work makes the following contributions: (1) it offers the first use case for the Contextual Matrix Profile (CMP) for adaptive anomaly detection in health care, specifically in a real-world remote health monitoring scenario; (2) it develops the multidimensional CMP and uses it to identify and score anomalous patient days; (3) it demonstrates the effectiveness of CMP-based anomaly scoring over state-of-the-art methods; and (4) it uses the CMP to discover biomarkers of anomalies using household movement data.

### Prior Work

#### Overview

Approaches to anomaly detection can be broadly categorized as statistical, distance-​based, reconstruction-​based, domain-​based or decision boundary–​based, information-​theoretic, and graph-​based [11]. Many approaches in the literature use combinations of techniques such as visual, knowledge-based, and machine learning approaches. We highlight how some of these techniques have been applied to anomaly detection in remote health monitoring scenarios.

#### Statistical Methods

Statistical thresholding is a popular approach to finding point anomalies. A National Institutes of Health–funded pilot study used statistical thresholding to generate alerts for UTI and offered early interventions for 37 older adult participants, some with Alzheimer disease, residing in apartments equipped with motion, pressure, and temperature sensors [12]. Clustering-based techniques were used in the study by Mori et al [13] to detect anomalies in the timing and duration of different activities. Statistical methods typically ignore the multivariate nature of anomalous events and can generate numerous false positives [14].

#### Machine Learning Approaches

Using early data from our Minder study, Enshaeifar et al [15] used a Markov chain to model activity sequences along with an entropy rate to quantify the regularity of an individual’s patterns in their day-to-day life. They used a training set to construct the Markov model and a verification set to define a confidence threshold for deviations [15]. Novák et al [16] detected anomalies such as long periods of inactivity, unusual presence, and changes in daily activity patterns using a combination of self-organizing maps for activity classification followed by a Markov model for next activity prediction. The limitations of the Markov approach include the inability to address parallel activities, activities that involve the same event with different probabilities, and scalability issues [17,18].

Arifoglu and Bouchachia [5] explored convolutional neural networks to capture temporal and spatial representations of activity and detect abnormal behavior related to repeating activities, sleep disruption, and confusion. Sensor data were sliced into time windows, and activities were labeled via sequence labeling to train convolutional neural networks that could detect deviations from normal daily life sequences. Supervised learning and interpretability are some limitations of this approach.

Akl et al [19] used signal processing with machine learning algorithms to detect mild cognitive impairments in older adults. They used sensors to extract the average, probability density, and trajectory of measures over sliding windows of sensor data as input to support vector machines and random forest classifiers to assess cognitive status. This approach requires training data annotations for cognitive status and has missing data issues in time windows.

Jakkula et al [20] considered the problem of anomaly detection based on temporal relationships. They expressed relationships between temporal events based on temporal logic, such as before, after, meets, overlaps, and contains, and used these to identify frequently occurring relationships between them. Adopting a probability-based model based on prior evidence from an inhabitant’s history, they reported low-probability events as anomalies. The study acknowledges that hundreds of sensors must be used to identify temporal relations at a granular level. It also requires a large training data set that must be updated to capture changing patterns.

Using data from our own remote monitoring study, Palermo et al [21] developed a supervised long short-term memory network to analyze the risk of agitation episodes in people with dementia using environmental, physiological, and sleep data. They used weak learning and label augmentation to address noise and class imbalance. In another Minder study, Li et al [22] adopted a semisupervised machine learning approach to predict the risk of UTI in people with dementia using environmental and physiological data. A convolutional autoencoder was used to learn a representation of the unlabeled sensor data. The encoder was used to extract the corresponding features from a smaller set of positively labeled data, which were then used to train a supervised classifier—a probabilistic neural network with a fully connected layer. Although this model is robust and learns continually, it approximates sensor data using Lagrangian approximation, requires interpretability, and takes a generalized versus patient-specific approach to detecting UTIs.

In the study by Paudel et al [23], the authors used unsupervised graph-based anomaly detection to identify cognitive health decline in older adult residents living in smart homes. They transformed motion sensor data from raw sensor log files into individual activity graphs and performed anomaly detection based on the normative pattern derived from the minimum data length principle [24]. This study used cohort-wide thresholds instead of the users’ own thresholds.

#### Visual Approaches

Visualization of activity density is another intuitive way of detecting anomalies in movement data. The study by Gupta et al [25] describes how unsupervised learning can be used to discover activity patterns in unlabeled data from passive infrared (PIR) sensors. In this work, user activity data were visualized and tracked through Uniform Manifold Approximation and Projection, whereas kernel density estimation was used for automatically extracting periods of dense sensor activity. Although Uniform Manifold Approximation and Projection plots are useful in informing daily patient-carer interactions, they are not readily interpretable, and this approach does not provide an anomaly score. Heat maps have also been used in conjunction with deep learning techniques to determine the probability of agitation- or UTI-related anomalies. In the study by Li et al [26], hourly heat maps based on raw sensor data were encoded via positional encoding to extract relevant time steps that were then passed into a long short-term memory model to extract relevant data and into an attention-based model to make predictions. This method uses supervised learning and, as is common with deep learning models, is computationally expensive and requires sufficient training data for accurate risk analysis and predictions*.*

#### Matrix Profile for Anomaly Detection

Research on real-world applications of Matrix Profile (MP)–based anomaly detection is scarce. Lin et al [27] used an early version of the MP to detect discords in electrocardiogram time series. More recently, researchers have used MP for web-based anomaly detection in IT operation time series [28]. In the study by Steenwinckel et al [29], researchers used an MP with knowledge-driven algorithms to create an interpretable system for sensor monitoring in the railway domain. Nieves Avendano et al [30] used MP with clustering for web-based anomaly detection and event prediction based on acoustic emission sensors that relay information about the mechanical conditions of a cold-forming manufacturing line. This method is robust to noise, missing values, and irregular sampling.

The CMP has been shown to be more flexible and effective than the MP in 2 curated non–health care web-based data sets where the authors showed how the CMP can be used to detect more subtle anomalies in addition to those detected by the MP [31].

**Figure 1:**
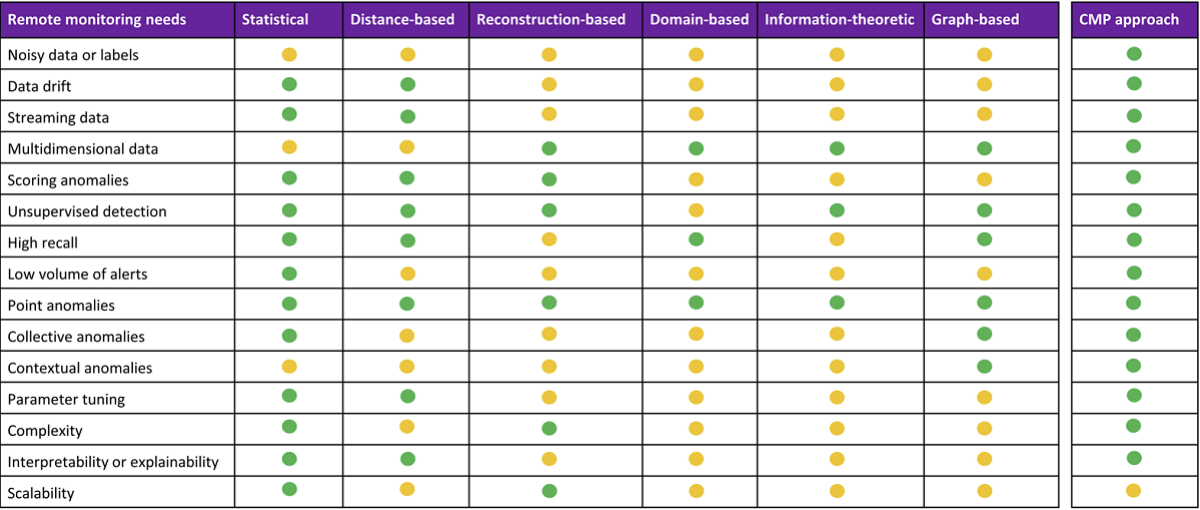
Suitability of anomaly detection techniques for remote health monitoring [11,32]. CMP: Contextual Matrix Profile.

In Figure 1 [11,32], we summarize the effectiveness of each technique framed in the context of remote health monitoring by evaluating the pros and cons of each technique presented in the survey literature. The CMP overcomes many of the drawbacks identified for distance-based methods and is well suited to remote health monitoring scenarios.

## Methods

### MP and CMP Preliminaries

#### MP Overview

The MP, detailed in the study by Yeh et al [33], is an unsupervised, state-of-the-art time series analysis technique that can be used for pattern detection, anomaly detection, time series segmentation, and change point detection. Its fast performance stems from the use of the fast Fourier transform for the *z*-normalized Euclidean distance computation. The algorithm is useful for both static data and incremental modeling of streaming values with limited slowdown on even very large and multivariate time series. In this section, we define the MP preliminaries relevant to anomaly detection in our smart home context.

#### MP Description

An MP *P* of time series *t* is a vector of the *z*-normalized Euclidean distances between each subsequence in an all-subsequence set *A* with its corresponding nearest neighbor or closest match within *A* (trivial matches excluded). Trivial matches are the set of subsequences around the query subsequence, which are likely to have a very small Euclidean distance from the query subsequence. This boundary is typically set to *m*/2, where *m* is the length of the subsequence.

#### Multidimensional MP

A *k*-dimensional MP of a multidimensional time series *t* with dimensionality *d* is a meta–time series that stores the *z*-normalized Euclidean distance between each subsequence and its nearest neighbor (the distance is computed using the *k*-dimensional distance function) [34]. In simple terms, the algorithm works as follows: (1) it stores the MP for each dimension (time series channel) in the subsequent rows of a 2D matrix, (2) the *k*-dimensional MP is computed by taking the average of the *k* lowest values in the columns of the matrix, and (3) the multidimensional MP is created such that row *k* (0≤ *k*< *m*) contains the *k*-dimensional MP. For implementation, we refer the reader to the STUMPY library tutorial [35].

The issues with the direct application of the MP are outlined in Textbox 1.

**Issues with the direct application of the Matrix Profile (MP).**

**Direct application issues related to the MP**

- The raw MP is noisy and does not give a clear indication of which discords are true anomalies.
- It is insensitive to amplitude variations and low in localization accuracy [28].
- The MP considers every subsequence for comparison with every other, which implies that the length of subsequence equals the level of granularity at which an anomaly may be identified. The two must be decoupled.
- An anomaly could be masked when its subsequence is close to another anomalous subsequence [28].
- The MP is hardwired to compute Euclidean distance. Although this has great advantages—complexity linear to the length of the time series, easy to implement, indexable, and parameter-free—it can also be sensitive to noise and exhibit misalignments in time [36].

#### CMP Overview

The CMP is a new flexible time series analysis technique based on the MP [31]. The CMP derives its motivation from the distance matrix calculations that are used to compute the MP. This section provides details on the CMP.

#### Context Window

**Figure 2:**
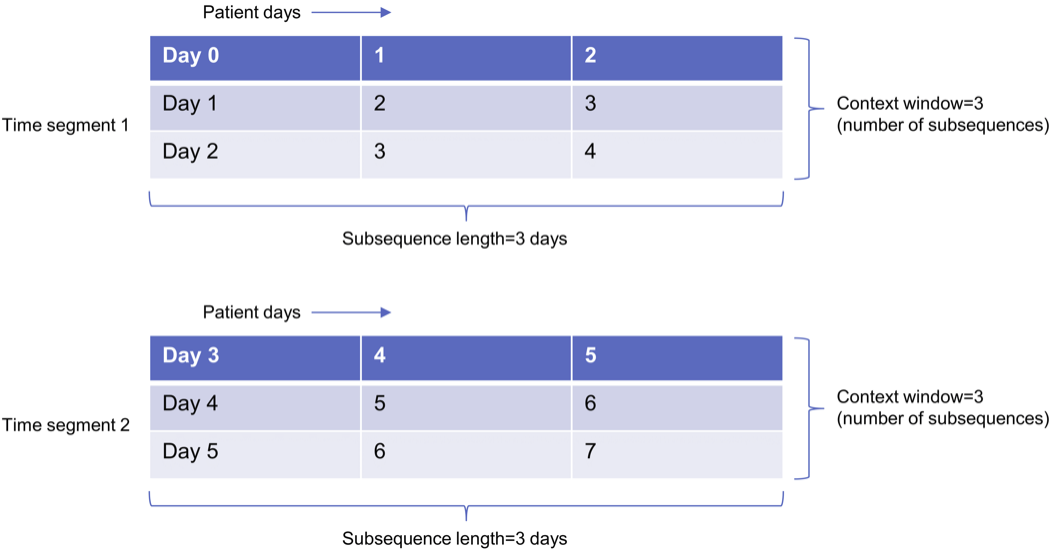
Contextual Matrix Profile contexts or time segments are blocks of time comprising a set of subsequences. Each context shown here is made up of 3 subsequences (context window=3), each subsequence being 3 days in length. We assigned anomaly scores to contexts instead of days.

It is the number of subsequences in a single time segment or region of interest. Given a patient data set, using a context window of 3 and a subsequence length of 3 (with no subsequences omitted), the patient data will be grouped into the time segments shown in Figure 2.

#### Context (or Time Segment)

It is a single time segment with a size equal to the context window and containing subsequences of length defined by the user. One cell in the CMP represents 1 “context” or time segment.

#### CMP Description

**Figure 3:**
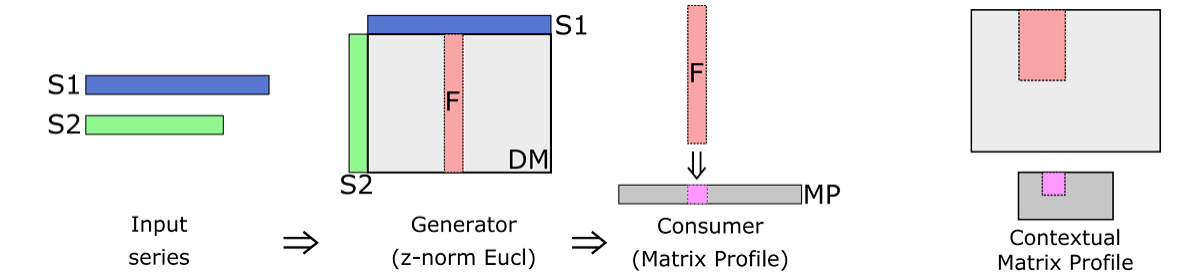
Matrix Profile versus Contextual Matrix Profile.

It is a configurable, 2D version of the MP that tracks the minimum distance between each context of subsequences in user-defined regions of the time series. First, the user (optionally) defines regions of interest for a given time series. They then determine the subsequence length and context window size. For instance, for a subsequence length of 3 days and a context window size of 3 days, the time series is divided into contexts, as shown in Figure 2. The CMP is formed by comparing the *z*-normalized Euclidean distance between each subsequence in one context and every subsequence in another context and selecting the minimum distance, which forms 1 cell in the CMP. Figure 3 highlights the difference between the MP and CMP. The MP comprises the column-wise minimum values in the distance matrix, whereas the CMP is created by taking the minimum over rectangular areas.

**Figure 4:**
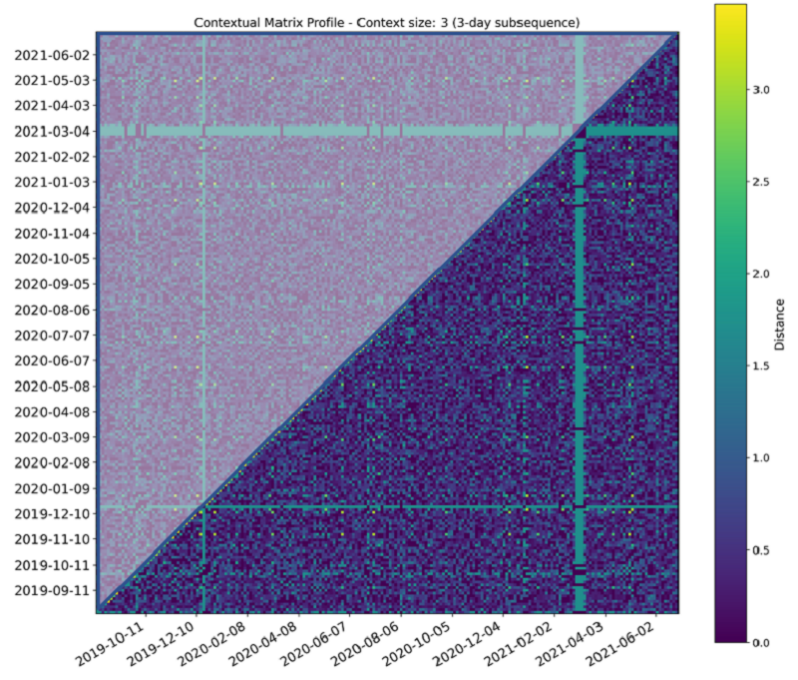
Each cell color codes the minimum distance between the time segments on the x- and y-axes. Green bands indicate anomalous activity or consistently large minimum distance from other time segments. The Contextual Matrix Profile is symmetric around the diagonal line.

The application of the MP idea to blocks of data instead of individual subsequences serves to aggregate and denoise the distance computation and extract useful patterns. Figure 4 shows the CMP for the late-evening daily bathroom activity for one of our patients. It serves as a visual overview of the consistency of activity and any break points.

#### Multidimensional CMP

We developed the multidimensional CMP based on the principle of a multidimensional MP. A *k*-dimensional CMP of a multidimensional time series *t* with dimensionality *d* is a meta–time series that stores the minimum z-normalized Euclidean distance between any subsequence in one context and any subsequence in another context, with the distance computed using the *k*-dimensional distance function, which is explained in the study by Yeh et al [34]. The algorithm works as follows. First, we stack the feature-specific 2D CMPs to obtain a 3D array. We then sort the array in ascending order using this feature dimension. This gives, for each context, the minimum distance values sorted in increasing order by feature. Now, we apply the method in the study by Yeh et al [34] to obtain the *k*-dimensional CMP. For *k*=0 (ie, 1D CMP), we query the first row of the *k*-dimensional CMP. This provides the lowest nearest-neighbor distance for each context based on a single feature. The lowest-scoring feature for a context may be different from the lowest-scoring feature for a different context. Similarly, for *k*=1 (or 2D CMP), we query the second row of the *k*-dimensional CMP, which, for each context, provides the lowest average distance based on 2 features. Again, the 2 lowest-scoring features for one context may be different from those for another context. The maximum value of *k* is the number of features minus 1 (*k* is zero-based).

**Figure 5:**
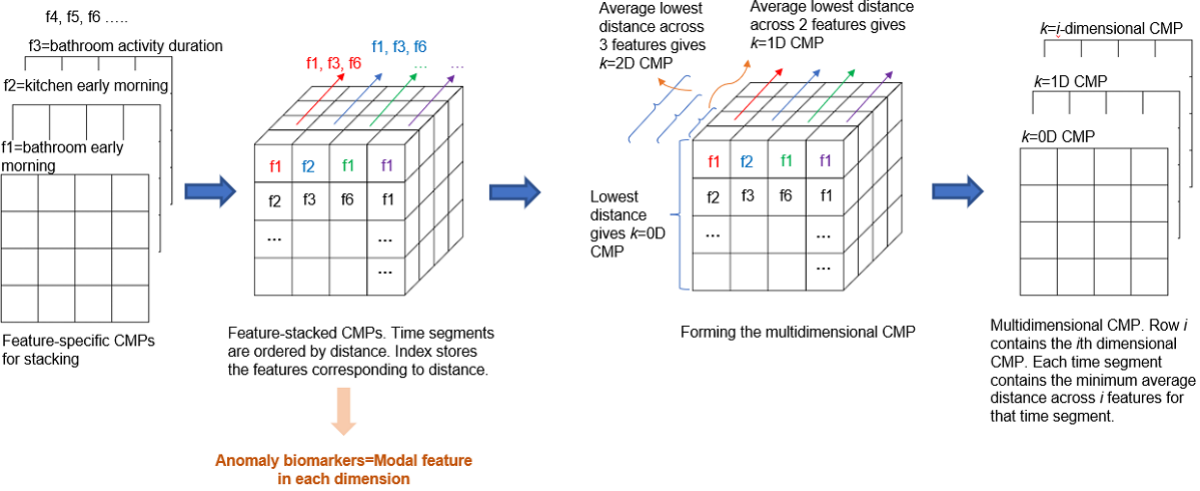
Multidimensional Contextual Matrix Profile (CMP) formation involves stacking feature-specific CMPs and then arranging each cell (time segment) in order of distance. The final multidimensional CMP is formed so that each cell in row i contains the average of the i+1 lowest distances for the cell.

The multidimensional CMP (Figure 5) is key to anomaly detection in our multidimensional data. The CMP offers advantages over the original MP (Textbox 2).

**Advantages of the Contextual Matrix Profile (CMP).**

**CMP advantages**

- It compares distance over a context instead of at a subsequence level, which is important for denoising the time series.
- The context size is configurable. In addition, the CMP allows for specific regions to be defined to detect patterns and anomalies, and the distance matrix need not be covered in its entirety.
- Other distance measures in addition to Euclidean distance can be used.
- The CMP offers an intuitive way of visualizing time series window regions and detecting anomalies.
- Anomalies cannot be easily masked, even if another similar anomaly has occurred elsewhere in the time series.

#### Anomaly Scoring With the CMP

**Figure 6:**
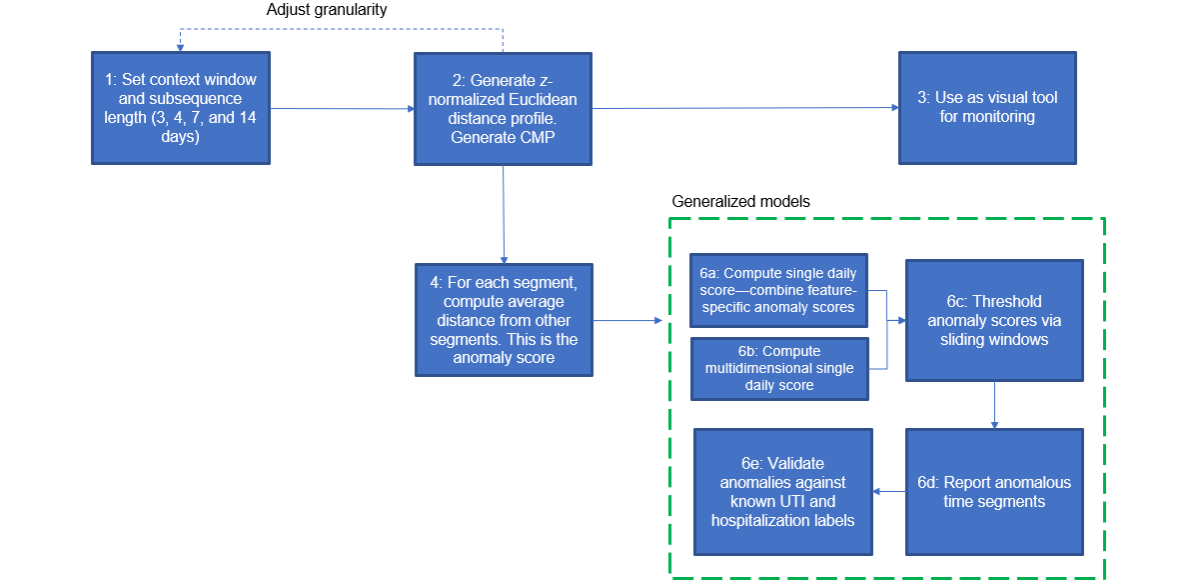
Contextual Matrix Profile (CMP)–based anomaly detection pipeline. UTI: urinary tract infection.

We used the anomaly detection pipeline (Figure 6) described in Textbox 3.

**Anomaly detection pipeline.**

**Pipeline for anomaly detection**

**Figure 7:**
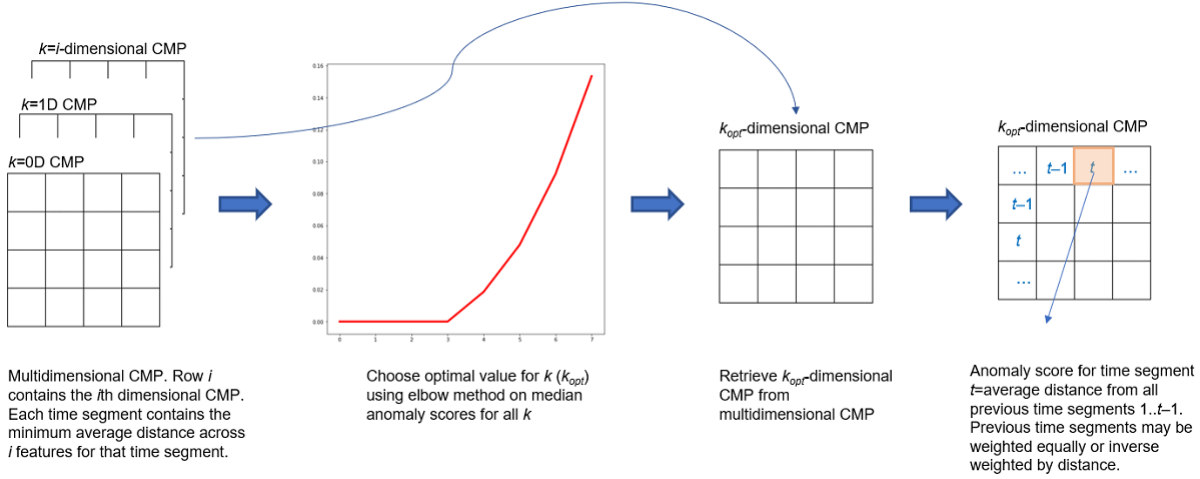
Multivariate anomaly scoring. Starting with the multidimensional Contextual Matrix Profile (CMP), we chose the optimal dimension for each patient by taking the median of their scores in each dimension and then selected the dimension at the inflection point. This optimal dimensional CMP was then used for distance-based anomaly scoring.

1. We decided on a suitable context window. We used a context size of 3 and a subsequence length of 3 days taking into consideration the need for maximum granularity, denoising, explainability, and time taken for the onset of an anomaly.
2. For each patient time series, we generated the z-normed Euclidean distance matrix for a self-join and then the Contextual Matrix Profile (MP) based on our context window setting.
3. We used the CMPs directly or adapted them for visualization and monitoring purposes.
4. We computed the anomaly score for each context. This is the average distance between the current context and contexts in the past. This step was repeated for every time series to obtain feature-specific anomaly scores.
5. The anomaly scores were used as inputs in different machine learning models trained for specific health events. This will be explored in future work.
6. The models were prepared to obtain a single-valued score for each time segment. We evaluated the following methods:
  - Combining feature-specific anomaly scores based on the sum of scores, median of scores, mean of scores, maximum of scores, and entropy-based weighting (the scores were combined based on the entropy of the underlying time series using inverse weighting; greater entropy implies lower weighting of the anomaly score obtained from using that time series). Two types of entropy measures were used:
    - Approximate Entropy (ApEn): ApEn approximates the exact regularity statistic Kolmogorov-Sinai entropy and reflects the predictability of a time series by exploring repetitive patterns in the data. It is applicable to noisy data sets [37]. It relies on the Heaviside function to define the similarity between 2 patterns. ApEn generates a unitless number from 0 (perfectly periodic) to 2 (noisy) [38].
    - Fuzzy Entropy (FuzzyEn): This also uses the Heaviside function, although similarity is evaluated by a fuzzy function that computes a membership coefficient ranging from 0 to 1. Consequently, in addition to the selection of *N* (length over which to compute entropy), *m* (subsequence length), and *r* (tolerance in terms of the number of SDs), FuzzyEn requires a fourth parameter, *n*, the gradient of the boundary of the exponential function used to assess similarity [39]. FuzzyEn provides a graded similarity instead of binary similarity between parts of the time series [37].
  - Multidimensional CMP-based scoring: We used the multidimensional CMP to generate the multivariate anomaly score for each context using 2 different settings for *k*:
    - *k*=auto: Here, we considered the optimal value of *k* when predicting true likely anomalies for a patient (Figure 7). To do this, we used the elbow method on each patient’s multidimensional CMP. Specifically, we computed the median distance in each of the *k*-dimensional CMPs for the patient and used the “kneedle” algorithm to automatically find the optimal value of *k* at which the inflection point occurred [40]. We then chose this optimal *k* row from the overall *k*-dimensional CMP to use this to extract the single-valued patient anomaly scores for each context. Once the “optimal” CMP was obtained, we scored each context in 2 ways:
      - Distance-weighted multidimensional CMP scoring: The anomaly score for a context was calculated as the inverse-weighted average of its nearest-neighbor distance from previous contexts. Thus, if a context is 3 hops in the past from the current context being scored, its distance is given one-third weight when calculating the anomaly score for the current context.
      - Equal-weighted multidimensional CMP: The anomaly score for a context was calculated as the simple average of its nearest-neighbor distance from previous contexts.
    - *k*=1: We took the CMP that is based on the top 2 features for each context.
  - We performed sliding window thresholding (7-, 14-, 21-, 30-, 60-, and 90-day windows) on the single context score using robust *z*, IQR, and quantile-based methods to predict true likely anomalies and report the best results.
  - The predicted anomalies were then “soft” validated against the anomaly labels available in the data set to compute recall.

### Data Set Description and Preprocessing

#### Data Set

The data for our study came from an ongoing real-world remote health care monitoring study (the “Minder” study mentioned in the *Introduction* section) of 102 people living with dementia at home and supported by their carers in England, United Kingdom. This includes 51% (52/102) men (mean age 81.94, SD 6.34 years) and 49% (50/102) women (mean age 80.80, SD 15.76 years). Movement data are captured via PIR sensors installed in different parts of the home—hallway, bathroom, bedroom, lounge, and kitchen—that are triggered passively based on movement throughout the day. There are door sensors, smart plugs for appliances, light and temperature sensors, a sleep mat, and physiological data recorders as well. We considered only PIR data in this study as they are the least missing, most reliable, and available with the finest granularity across the cohort. Physiological data are currently self-reported by the person living with dementia or their carer once or twice a day and with greater missingness, which would require imputation. Sleep data are sparse for this cohort.

In our study, we focused on the 15 patients with dementia who had had at least one clinically validated incidence of UTI. This included 53% (8/15) men (mean age 85.13, SD 5.57 years) and 47% (7/15) women (mean age 82.86, SD 6.79 years). Of these 15 patients, 7 (47%) had also experienced ≥1 hospitalization event. Altogether, we had 31 UTI and 10 hospitalization labels across a total of 9363 patient days, making up approximately 0.44% (41/9363) of the overall data set. The UTI labels were manually annotated after validation by the clinical monitoring team using urine samples from patients. However, it is worth noting that older adult patients often present with atypical symptoms, making the differentiation of asymptomatic bacteriuria from symptomatic UTI challenging [6]. Moreover, the start time of UTI and the duration of symptoms are not clearly defined. The list of hospitalization events was collated based on information from general practitioners. It included the date of hospitalization and, in some but not all cases, the cause of hospitalization.

#### Preprocessing

##### Daily Aggregation

Household movement data captured via PIR motion sensors were first aggregated daily to reduce noise, as hourly counts can vary widely from one day to the next, and the high granularity and variation make anomalies less discernible. We ignored consecutive firing events from the same sensor, considering the first firing event to compute the duration at the previous location and the last firing event to compute the duration at the current location. Any consecutive sensor firings from the same sensor between the first and last firing were ignored, thus reducing redundancy and noise.

##### Feature Engineering

To capture different types of movement-related information, we calculated the features outlined in Textbox 4 for the daily activity data across the various locations—bathroom, bedroom, kitchen, lounge, and hallway.

**Movement-related features.**

**Feature and description**

- Location count: this is the daily count of sensor firings for each location.
- Location early-morning count: this is the count of sensor firings between midnight and 6 AM on the current day.
- Location late-evening count: this is the count of sensor firings between 6 PM and midnight on the previous day.
- Location duration: this is the daily total number of minutes spent at each location.
- Location hourly movement change: this is the Wasserstein distance between the hourly sensor distribution at a location on the previous day with that on the current day; a larger Wasserstein distance implies a greater change in hourly pattern from one day to the next; this measure is robust to different motion densities across patient households. The Wasserstein distance or “earth mover” distance is a single explainable metric that measures the approximate *minimal work* required to move between 2 probability distributions, where “work” can be loosely defined as the product of how much of the distribution’s mass moves and the distance by which it must be moved [41]. Unlike other measurements such as L2, Kullback-Leibler divergence, and Jensen-Shannon divergence, the Wasserstein distance is sensitive to geometry [42].

##### Feature Selection

Similar to the study by Skubic et al [43], we applied the methods outlined in Textbox 5 to select the features for anomaly detection.

The simplified list of features included bathroom Wasserstein distance, hallway Wasserstein distance, lounge Wasserstein distance, bathroom early-morning and late-evening activity, kitchen early-morning and late-evening activity, bedroom early-morning and late-evening activity, bedroom activity duration, and bathroom activity duration.

**Methods for selecting features for anomaly detection.**

**Anomaly detection feature selection methods**

- Domain knowledge: the study by Pevný [44] showed that detectors using only features that explain anomalies had equal or better performance than detectors using all features. Erratic bathroom activity can strongly suggest urinary tract infection [45], and therefore, we captured daily changes in bathroom activity. Similarly, disturbed sleep, agitation, and wandering are common characteristics in patients with dementia [46,47]. Hence, we included daily changes in the hourly distribution of bedroom, hallway, kitchen, and lounge activity. These features help capture unusual daytime and night-time activity across locations and follow recommendations by clinical researchers in a similar study supporting the modeling of health decline with behavioral biomarkers [43].
- The significant online discords technique was used to find the common features that are associated with the highest median recall value for urinary tract infection and hospitalization using cross-validated data from our patient cohort [48].
- We eliminated redundant variables based on the correlation between the features.
- We also eliminated duration-related features for communal spaces such as hallways, living rooms, and kitchens, where distinguishing between patient and carer activity is difficult as of yet.
- The variables that were robust to differences in activity levels across households were retained.

### Experiments

We conducted our experiments on the household movement data of 15 patients selected from the ongoing Minder study, which had 31 UTI and 10 hospitalization labels across a total of 9363 patient days. All experiments were run on a 64-bit Intel i7-8700K central processing unit, 3.7 GHz Windows 10 machine with 32 GB of RAM.

Our experimental settings are listed in Table 1. For each anomaly-scoring model, we experimented with every combination of window size, IQR threshold, robust *z* threshold, and quantile threshold and reported the best results obtained.

**Table 1:** Experimental parameters considered in this study.

Setting			Values
Context window			3
Subsequence length (days)			3
Window sizes for sliding window thresholding (days)			7, 14, 21, 30, 60, and 90
IQR threshold			1.0 and 1.2
Robust *z* threshold			1.65, 1.8, 3, and 4
Quantile threshold			0.95, 0.96, 0.97, and 0.98
**Entropy-based methods**			
	N (data size)	500	
	r (SD tolerance)	0.2	
	m (subsequence length)	7	
Soft buffer for label validation (days around actual label)			−10 to +7

### Model Evaluation

We used the data from our 15 patients to evaluate multidimensional CMP-based anomaly scoring relative to univariate methods and 3 other popular modern, parameter-free, and interpretable methods in the literature: Angle-Based Outlier Detection (ABOD), Copula-Based Outlier Detection (COPOD), and Lightweight Online Detector of Anomalies (LODA). To be suitable for use in an unsupervised, streaming scenario, we used only historical data at each time point.

ABOD measures the variance of the angle (cosine) spectrum of the data points weighted by the corresponding distances. ABOD works on the principle that if the spectrum of the observed angles for a point is small, other points will be positioned only in certain directions. This means that the point is positioned outside of some sets of points that are grouped together, implying that the point is an outlier [49]. COPOD is inspired by copulas for modeling multivariate data distributions. COPOD first constructs an empirical copula and then uses it to predict the tail probabilities of each given data point to determine its level of “extremeness.” The outlier scores produced by COPOD measure the likelihood of a point relative to the other points in the data set. The method outputs a “dimensional outlier graph” that provides insights into outlier subspaces or features for a given outlier point [50]. LODA comprises a collection of *k* 1D histograms, each approximating the probability density of the input data projected onto a single projection vector. Projection vectors act to diversify individual histograms, which enables the ensemble system to improve the performance of a single detector. The complexity of LODA is linear with respect to the number of training samples and the dimension of the input space [44].

We used 3 thresholding criteria for scores (Textbox 6).

To determine how competitive CMP-based anomaly scoring is in identifying anomalies in real-world remote monitoring data for patients with dementia, we report the measures shown in Textbox 7 for each model.

An anomaly is assumed to be correctly identified if the predicted date is within the soft buffer of the labeled date of anomaly. For transparency, we report both the average recall and patient-wise recall. From a clinical perspective, this measure is a direct indication of a model’s effectiveness.

When choosing between models, a clinician will likely choose a model with a higher average recall, as the cost and inconvenience of false alerts in our scenario are considerably less than the cost of missing a real anomaly.

**Thresholding criteria for scores.**

**Criteria for thresholding of scores**

- The robust *z* thresholding or Median Absolute Deviation method is less influenced by outliers and is used to calculate a modified *z* score that quantifies the anomaly score in terms of SD units away from the median [51].
- Tukey or IQR thresholding uses the IQR of anomaly scores in the sliding window as the basis for thresholding. Any value greater than the third quartile+ *x* times IQR is deemed anomalous, where *x* is the IQR threshold from Table 1.
- Quantile-based thresholding uses a fixed percentile of anomaly scores as the basis for thresholding taken from Table 1.

**Model evaluation.**

**Model evaluation measures**

- Number of patients with >33% recall: given that the average patient had only 3 validated anomalies, we ranked the models based on how many patients had greater than one-third of their anomalies correctly identified. This makes it transparent whether the model is just effective for a small proportion of patients or across the cohort.
- Average recall: this is the average percentage recall across the 15 patients, where recall=true positives or all known anomalies in the data set.
- Average number of anomalies detected: to minimize false alerts made to the clinical monitoring team, lower is better.
- Average recall percentage versus anomalies raised: according to the study by Pimentel et al [11], effectiveness in novelty detection is based on the detection rate and the false alarm rate. The best model will demonstrate high recall together with a low number of anomalies raised.
- Precision: here, *precision* has little meaning, as outliers may result from different types of health indicators, sensor failures, visitors, pet activity, or rare unusual activities by the patient or carer, which are not labeled in our data set. Although we still report this metric, relative precision across methods is more meaningful.

### Digital Biomarkers

Digital biomarkers are consumer-generated physiological and behavioral measures collected through connected digital tools that can be used to explain, influence, or predict health outcomes [52]. The Food and Drug Administration-National Institutes of Health “Biomarkers, EndpointS, and other Tools” classification for traditional biomarkers classifies their use into the following categories: susceptibility or risk determination, diagnostic use to detect and confirm the presence of a condition of interest, monitoring of the status of a condition, prognostic use to identify likelihood, recurrence or progression of a condition, predictive use, and measurement of response through exposure to a medical product or agent [53]. We envisage these biomarkers of anomalies to be used for susceptibility determination and assistance with diagnosis, prognosis, and prediction of UTI or another adverse clinical event.

The creation of the multidimensional CMP involves the intermediate step of combining feature-specific CMPs such that each context is arranged in ascending order of the feature-wise nearest-neighbor distance. This implies that if we simply keep track of the ordered set of features for each context in the ordered stacked CMP, we can discover the most common contributing feature in each of its dimensions. The modal feature in the 0th dimension will be the most important biomarker associated with the patient’s anomaly score. The modal feature in the first dimension will be the second most important biomarker and so on. Subsequently, by looking across the ordered stacked CMPs for the entire cohort, we can determine the generalized top *k* important biomarkers.

### Ethics Approval

This study received ethics approval from South East Coast Surrey National Health Service Research Ethics Committee (Health Research Authority); Technology Integrated Health Management Research Ethics Committee Reference: 16/LO/1802; Integrated Research Application System ID: 211318.

## Results

### Model Evaluation

We report the best results for each type of univariate and multivariate model (Table 2).

All models could correctly identify more than one-third of the known anomalies for two-thirds of the patients in the study. Of these, the multidimensional CMP with equal-weighted context (at window size=7 days, robust *z*=1.65, and *k*=1) yielded >33% recall for 100% (15/15) of the patients. Other CMP-based methods showed similar recall for up to 93% (14/15) of the patients. This highlights the strong support for multidimensional CMP as an anomaly detection tool for this cohort.

We also measured how many anomalies were raised by each of the models across the 624 average patient days in our study. As shown, the maximum number of alerts raised by any of the CMP-based models was only approximately 34 or 5.4% (34/624) of patient days. Our best-performing CMP model raised approximately 32 alerts, which is, on average, 5.1% (32/624) of patient days. Note that there were, on average, 3 labeled anomalies in our data set per patient; however, as emphasized previously, the annotated anomalies covered only UTI and hospitalization, and our models were designed to pick up on any anomalous activity.

The average recall, when viewed together with the total detected anomalies, provides a holistic view of performance, as it is easily possible to obtain a top-performing model by identifying an extraordinarily high number of anomalies. The overall best model is one that demonstrates high recall but a low number of raised anomalies. It is clear that the multidimensional CMP with equal-weighted contexts at window size=7 days, robust *z*=1.65, and *k*=1 offers the best-balanced performance, raising only 32 alerts over a 624-day patient journey on average. ABOD yields relatively low recall, whereas LODA and COPOD yield high recall but with a higher number of alerts raised than our best-performing model.

**Table 2:** Model performance (N=15).

Model	Patients with >33% recall, n (%)	Anomalies raised, mean	Recall (%), mean	Precision, %^a^
LODA^b^ (w=7; IQR 1.2)	14 (93)	37.8	85.7	6.2
Sum of CMP^c^ scores (w=7; quantile 0.97)	14 (93)	33.1	84.7	7.0
Mean of CMP scores (w=7; quantile 0.97)	14 (93)	33.1	84.7	7.0
Equal-weighted multidimensional CMP (w=7; *k*=1; robust *z*=1.65)	15 (100)	32.1	84.3	7.2
COPOD^d^ (w=7; quantile 0.95)	13 (87)	36.8	79.1	5.9
ABOD^e^ (w=21; quantile 0.95)	13 (87)	30.0	77.7	7.1
Distance-weighted multidimensional CMP (w=14; *k*=0; robust *z*=1.65)	14 (93)	33.7	76.7	6.2
ApEn^f^-weighted CMP scores (w=7; quantile 0.97)	12 (80)	29.1	69.9	6.8
Median of CMP scores (w=7; quantile 0.97)	12 (80)	30.8	68.4	6.1
Fuzzy entropy–weighted CMP scores (w=7; quantile 0.97)	10 (67)	27.7	65.5	6.5
Maximum of CMP scores (w=7; quantile 0.97)	10 (67)	24.8	57.9	6.4

^a^We have mentioned previously that it is more meaningful in this context to look at relative precision across methods and not at absolute precision.

^b^LODA: Lightweight Online Detector of Anomalies.

^c^CMP: Contextual Matrix Profile.

^d^COPOD: Copula-Based Outlier Detection.

^e^ABOD: Angle-Based Outlier Detection.

^f^ApEn: Approximate Entropy.

### Digital Biomarkers

**Figure 8:**
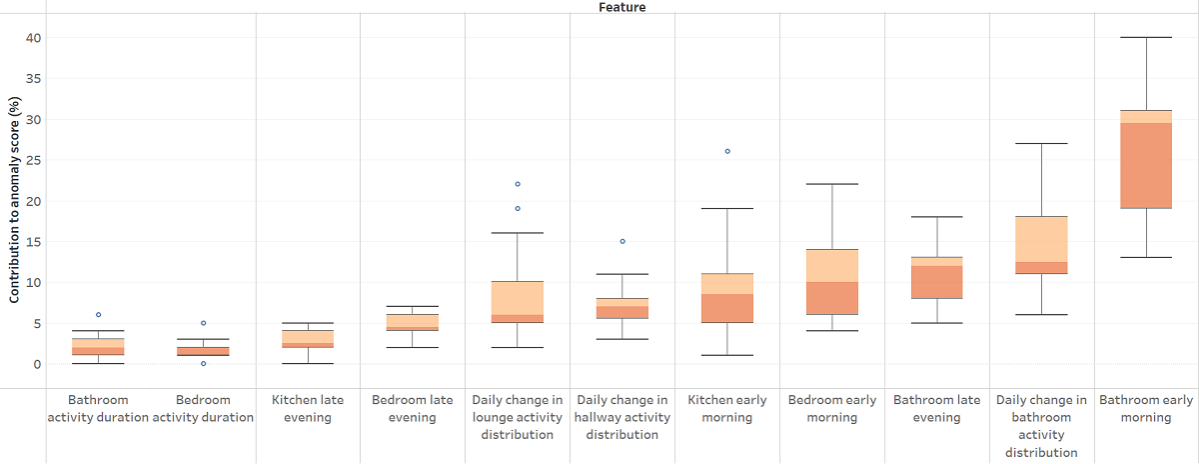
Top contributing digital biomarkers of anomalies. Early-morning bathroom activity had the largest median contribution of approximately 30% to the overall anomaly score.

As seen previously, the multidimensional CMP for a patient can be used to discover the important digital biomarkers of anomalies. In Figure 8, we show the magnitude of the contribution of significant features toward the anomaly score across the cohort.

We discovered that early-morning (midnight to 6 AM) bathroom activity was the single largest contributor to the anomaly score by a wide margin, with a median value of approximately 30% for this cohort. This validates the findings in the literature that unusual bathroom activity is a clinically significant feature of UTIs [12,54], which comprises three-quarters of the anomalies in our labeled data set. Patient-level investigation showed this to be the top biomarker for 60% (9/15) of the patients. Late-evening (6 PM to midnight) bathroom activity also had a contribution of 12%. Both factors correlate with sleep disruption, which is commonly seen in people living with dementia. Unusual bedroom and kitchen activity in the early hours of the morning are also among the significant contributors to anomaly scores, pointing to wandering and disturbed sleep seen in dementia.

**Figure 9:**
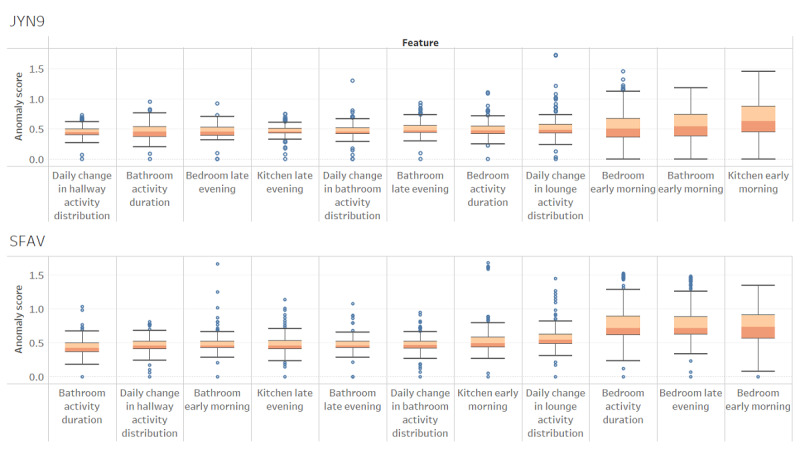
Univariate anomaly score distribution for 2 patients.

The multidimensional CMP also provides intuitive insights into patient-specific anomalies. Figure 9 shows the anomaly scores associated with 2 patients, ordered by the median anomaly score.

For patient JYN9, unusual early-morning kitchen activity was the prime biomarker of anomalous activity, where we also see the largest variance in anomaly scores. For patient SFAV, unusual bedroom activity was the largest contributor to their anomaly score. These figures indicate different anomaly patterns in the 2 patients, presumably agitation and wandering in the first patient and sleep disruption and shifting bedroom activity over time in the second patient. We can envisage an anomaly detection dashboard to provide such insight to clinicians to enable them to target interventions as needed.

**Figure 10:**
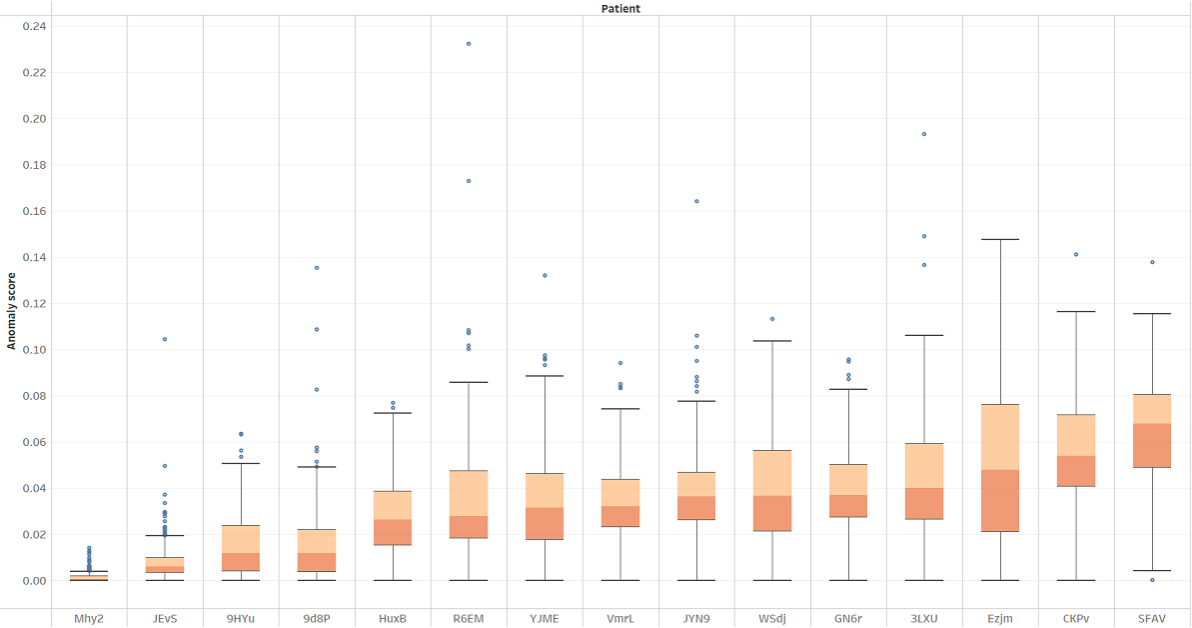
Multivariate anomaly score distribution for all patients.

We can also use the standardized anomaly scores to look at a cross-patient view (Figure 10), where we see the cross-cohort variation in multivariate anomaly scores using the patients’ own optimal *k*-dimensional scores. It would be interesting to investigate patient differences in relation to their cognitive scores.

## Discussion

### Principal Findings

Early identification of anomalies in patients living with dementia provides a window of opportunity for early intervention before a major health event occurs. This implies improved health outcomes, reduced health care costs, continued independence, and better quality of life [43]. In this study, we developed an MP-driven approach for anomaly detection and evaluated its use in a real-world study of sensor-based remote monitoring of people with dementia. We developed the multidimensional CMP to model patient household activity from sensor data and used the average Euclidean distance between activities in time segments as the basis for generating a single anomaly score. The CMP-based approach overcomes the issues with traditional distance-based anomaly detection techniques, namely, degradation because of noise, high alert rate, and identification of local novelty. Our experiments show that multidimensional CMP-based anomaly detection performs better than other comparable fast, modern, exact, and parameter-free unsupervised techniques for anomaly detection. It is well suited to real-world remote monitoring data characterized by noise and incomplete labeling and is additionally useful as a visual tool for operational monitoring, also lending itself to the discovery of personalized and cohort-wide digital biomarkers. The personalized model for each resident comes uniquely from their own sensor data patterns [43]. These aspects make CMP-based anomaly detection clinically significant, interpretable, and immediately usable, freeing up clinicians’ valuable time from having to annotate patient activity. The CMP is domain agnostic and can be easily extended to different types of health care data and domains. To the best of our knowledge, our work is the first real-world use case of CMP in health care anomaly detection.

Our experimental parameters were selected to be clinically relevant. A context window of 3 and a subsequence length of 3 were selected for maximum granularity, noise resistance, and suitability for anomalies such as UTI, where a 3-day pattern is more likely to throw up anomalous behavior than a more granular daily analysis; it typically takes 3 to 8 days for a UTI to present [55]. The context window and subsequence length can be easily configured to investigate anomalies at different levels of granularity, such as 7 days, 2 weeks, or 1 month. Similarly, we used 7-, 14-, 21-, 30-, 60-, and 90-day windows to threshold anomaly scores. Time segments such as these follow typical human patterns of behavior and are easily understood by clinicians. Threshold values for IQR, robust *z* and quantile-based thresholding, and entropy parameters were chosen to mirror values widely used in the literature. The soft buffer for label validation (−10 days to +7 days of actual anomaly label) reflects the issue of weak labeling because of noise and inaccuracy because of manual labeling, the time it takes for a UTI to develop and be clinically diagnosed, and the need to catch anomalies early. Dau and Keogh [56] used a similar evaluation technique for weakly labeled data. We chose ABOD, COPOD, and LODA for comparison with CMP-based methods as they are similarly high-performing, parameter-free, interpretable, unsupervised anomaly detection techniques relevant to a streaming data scenario such as remote health monitoring.

Our evaluation methods were also designed to be simple, transparent, and clinically meaningful. A good method must demonstrate high cross-cohort average sensitivity but also high sensitivity for individual patients while raising minimal alerts. Therefore, we report the overall sensitivity, patient-wise sensitivity, average number of anomalies raised, and recall versus anomalies raised, which provides a rounded measure of performance. Although we report the precision for each model, it must be noted that we only considered 2 types of labels—UTI and hospitalization—whereas our models identify all types of anomalies in household movement data, many of which cannot be validated using existing labeled data. For this reason, a low absolute precision is to be expected, and the relative precision offers a better indication of the cross-model performance in our study. Moreover, the 5% alert rate is an acceptably low rate as these alerts may have critical implications for the health of people with dementia. This was also the approach taken by Rantz et al [12], and our clinical care teams already conduct weekly check-ins with the patients.

Our results show that for our top-performing models, the optimal sliding window size for thresholding is 7 to 14 days (ie, 1-2 weeks). This makes intuitive sense as an “anomaly” regarding human behavior can be perceived as a break in their recent routine. This was also clinically validated in the study by Skubic et al [43], where clinicians recommended a 2-week moving baseline for sensor data comparison and thresholding to balance capturing sudden and gradual health changes. A short sliding window has the added advantage of being robust to variations in patient characteristics and environmental conditions. However, the ABOD technique is highly sensitive, requires sufficient data to capture true outliers, and performs best with a minimum look-back of 21 days. This behavior of increasing the sample for better performance of ABOD was also validated in the study by Domingues et al [57].

We make 3 striking observations. First, the top-performing model in terms of balancing cohort-wide sensitivity and raised anomalies was based on *k*=1 (ie, it considers only the top 2 contributing features for a patient). This implies that a reliable anomaly detection model based on patient activity can be simple, lightweight, easily interpretable, and generalizable. Second, univariate models derived from combining feature-specific CMPs via simple aggregation (ie, sum and mean of feature-specific anomaly scores) achieve both high recall and low volume of alerts. They are, in fact, closer in performance to the best-performing multivariate CMP model than more established high-performing models such as LODA, ABOD, and COPOD and other complex ways of combining univariate scores such as entropy-weighted scores. This shows once again that simple, interpretable models can generalize and perform competitively. Third, it is surprising that an equal-weighted time segment–scoring approach achieves considerably better recall than distance-weighted time segment scoring. We would expect that by emphasizing more recent time segments over past time segments, we might obtain an anomaly score that is reflective of a true anomaly in the current time segment. However, this appears to not be the case in this study. We aim to explore different ways of weighting previous time segments to confirm whether this behavior was because of the specific distance-weighting logic used or a more general finding.

Digital biomarkers are an incredibly useful artifact of our method. They tell us what kind of household activity was responsible for the anomaly at a specific period. Furthermore, looking across a patient’s timeline, we can find the single most common activity or feature that contributed most frequently to the anomaly score in the time segments overall (ie, a digital biomarker of their anomalous behavior). We discovered that cohort-wide, early-morning (midnight to 6 AM) bathroom activity was the most common digital biomarker of anomalous behavior (9/15, 60% of the patients), followed by late-evening bathroom activity and early-morning bedroom and kitchen activity. These findings quantitatively validate observational studies of patients with dementia, where agitation, wandering at unusual hours, and unusual bathroom activity, particularly early-morning and late-night bathroom activity, were observed, especially in patients with dementia experiencing a UTI [14,20,45,46]. Finally, our method can provide a ranking of digital biomarkers for anomalies at the time segment, patient, and cohort levels. This outcome makes CMP-based anomaly scoring independently useful for clinical monitoring and for querying and validating digital biomarkers.

There are a few notable differences between this work and existing published research based on the Minder study. First, published works have used a variety of supervised and semisupervised machine learning methods to detect or predict targeted health conditions such as agitation and UTI (one study used unsupervised learning to isolate anomalous movement patterns via clustering). As such, the models were trained with data from the subset of patients clinically validated to have the specific health condition in their trajectory. In contrast, our work evaluated a lightweight, unsupervised, and parameter-free approach to detect general anomalies based on household activity data. It requires no training data but is validated on data from patients who have experienced one or more UTIs and hospitalization events. Second, existing studies incorporate patient physiological data and household appliance use in addition to household activity. We currently use only household movement data. Third, existing studies rely on either fixed training data or periodically refreshed training data, whereas our approach was designed to work in a streaming environment, implying that our daily detection and alerting algorithm uses the information in the patient timeline up to the current day. Fourth, our algorithm is patient data driven rather than cohort data driven. This means that we evaluate the average recall by considering the algorithm’s performance on individual patient data. In contrast, published work takes a cohort-wide or patient-blind approach to assess algorithm performance. These factors should be collectively considered when comparing our work with other Minder-based research.

The CMP-based approach is ideally suited to anomaly detection applications where data and labels are characterized by real-world noise and annotated training data required for supervised learning may not be available because of resource constraints or in a streaming data scenario, as well as where the distinction between normal and anomalous data is not clear-cut. This includes sensor-based remote health monitoring in a variety of industrial, urban, and health care settings. The CMP-based approach excels at zooming out and focusing on temporal patterns at configurable time scales. It is also designed with personalization in mind, which makes it especially relevant for health care, where patterns of similar anomalies or the same disease can present differently in different individuals. It is ideal for situations in which explainability is key for operational monitoring teams.

The CMP-based approach may not be ideal for applications that prioritize sensitivity over interpretability. It is also not the best tool for data that have a well-defined, well-understood pattern, such as electrocardiogram data, or where noise levels are low or the distinction between normal and abnormal data is clearly understood. Finally, the CMP pipeline would need to be augmented with feature reduction methods for it to scale to high-dimensional data.

### Limitations

A limitation of the anomaly detection method presented in this paper is that cross-sensor correlations were not considered. This will be investigated in future studies using interpretable machine learning. Second, our study ignored sensor data from the front and back doors. This omission was intentional as we were interested in detecting anomalies arising from significant changes in indoor household activity instead of those arising from out-of-home situations. In addition, front door and back door opening and closing are as of yet difficult to attribute to the person with dementia. Third, a system to distinguish the patient from other household members is needed to improve the robustness of anomaly detection models based on passive sensing. Fourth, to achieve finer granularity and lower latency than 1 “context,” the CMP-based anomaly detection model should be configured to ingest data hourly or at a higher resolution than 1 day. Fifth, we assume that an anomaly in a single time window can be deterministic of a complex health event. However, the presence of pets and visitors could also contribute to anomalies. To address this, we require not only a distinction between patient and carer but also a way of monitoring anomalies in subsequent time windows to correlate anomalies with health changes with high confidence. Finally, we will require a larger sample size to further validate our approach.

### Conclusions and Future Work

In this study, we developed a novel lightweight unsupervised anomaly detection pipeline based on the CMP and evaluated it in sensor-based remote health monitoring of patients with dementia. We combined univariate CMP scores in novel ways, developed the multivariate CMP, and tested it for identifying anomalous patient days via thresholding in sliding windows. We demonstrated CMP-based anomaly scoring to be more effective and generalizable than other comparable methods for unsupervised anomaly detection. Specifically, the multidimensional CMP based on a 7-day sliding window and using the top 2 contributing patient-specific features exhibits 84.3% recall with only 32 alerts over the average patient timeline of 624 days. In addition, we showed how the CMP can be used to uncover and explain digital biomarkers of anomalies at the time segment, patient, and cohort levels. Our study of 9363 days collected from 15 people living with dementia who had UTI and hospitalization events in their timeline showed that unusual bathroom activity in the early and late hours of the day is a prominent biomarker of anomalies across our cohort. This helps quantitatively validate observational studies of similar behavior in patients with dementia.

Our future work will focus on the following areas: adding physiological data to the anomaly-scoring pipeline, developing the CMP as a tool for effective visual monitoring of patterns and anomalies in data and accommodating other distance metrics in addition to Euclidean distance, validating the CMP on a larger patient cohort and different kinds of anomalies, and using machine learning methods to use CMP-based scores to classify different types of anomalies. We will also investigate seasonal effects and compare our method with other relevant anomaly detection methods. We plan to integrate our model into the Minder platform to raise alerts when anomalies are detected to enable the monitoring team to investigate the underlying sensor data and offer timely intervention to patients. Alerts that are validated as true will be recorded in the patient timeline and used to monitor the operational accuracy of our model.

## Appendix

Multimedia Appendix 1 Acknowledgment list for the UK Dementia Research Institute.

## Abbreviations

ABOD: Angle-Based Outlier Detection
CMP: Contextual Matrix Profile
COPOD: Copula-Based Outlier Detection
LODA: Lightweight Online Detector of Anomalies
MP: Matrix Profile
PIR: passive infrared
UTI: urinary tract infection

## Data Availability

Access to the complete data set requires ethics approval and is not currently available because of the sensitivity of the in-home monitoring data and privacy and security requirements. To inquire about the complete data set, a request with a statement of intent and use must be sent to Professor Payam Barnaghi, Head of the Machine Intelligence group at UK Dementia Research Institute Care Research and Technology Centre at Imperial College.
