# Supplementary material for: An Unsupervised Data-Driven Anomaly Detection Approach for Adverse Health Conditions in People Living With Dementia: Cohort Study
Source: JMIR Aging. 2022 Sep 19;5(3):e38211. doi: 10.2196/38211 (PMC9531007; doi:10.2196/38211)
Supplement: Multimedia Appendix 1 [file aging_v5i3e38211_app1.pdf]

Acknowledgement list for UK Dementia Research Institute (UK DRI) Care Research & Technology (CR&T) Centre publications using the MINDER core data set

The UK DRI CR&T senior management team have agreed the list below for standardised acknowledgement. All papers should include this full list.

Leadership and Management Infrastructure:

Centre Director: Professor David Sharp
Co-Director: Professor Payam Barnaghi
Centre Manager: Danielle Wilson
Health and Social Care Lead: Sarah Daniels
Project Managers: Mara Golemme and Zaynab Ismail, Imperial College London
Group Leaders: Professor David Sharp, Professor Payam Barnaghi, Professor Paul Freemont, Dr Ravi Vaidyanathan, Professor Tim Constandinou, Imperial College London
Professor Derk-Jan Dijk, University of Surrey

Groups: Behaviour and Cognition led by Prof David Sharp

Michael David MD
Martina Del Giovane
Neil Graham MD PhD
Naomi Hassim
Magdalena Kolanko MD
Helen Lai
Lucia M Li MD PhD
Thomas Parker MD PhD
Mark Crook Rumsey PhD
Paresh Malhotra MD PhD
Emma Jane Mallas PhD
Greg Scott MD PhD
Alina-Irina Serban
Eyal Soreq PhD
Tong Wu PhD

Biosensor Hardware led by Prof Timothy Constandinou

Alan Bannon PhD
Shlomi Haar PhD
Charalambos Hadjipanayi
Ian Williams, PhD
Ghena Hammour
Bryan Hsieh
Adrien Rapeaux PhD
Maowen Yin

Robotics and AI interfaces led by Dr Ravi Vaidyanathan

Maria Lima
Maitreyee Wairagkar PhD

Machine intelligence led by Professor Payam Barnaghi

Nan Fletcher-Lloyd
Hamed Haddadi PhD
Valentinas Janeiko
Anna Joffe
Samaneh Kouchaki PhD
Viktor Levine
Honglin Li
Amer Marzuki
Francesca Palermo
Mark Woodbridge
Yuchen Zhao PhD
Alexander Capstick
Severin Skillman

Point of care Diagnostics led by Professor Paul Freemont

Loren Cameron PhD
Michael Crone PhD
Kirsten Jensen PhD
Martin Tran
Thomas Adam

Sleep and Circadian led by Professor Derk Jan Dijk

Anne Skeldon, PhD
Kevin Wells, PhD
Ullrich Bartsch PhD
Ciro Della Monica PhD
Kiran GR Kumar PhD
Damion Lambert
Sara Mohammadi Mahvash PhD
Thalia Rodriguez Garcia PhD
Vikki Revell PhD
Giuseppe Atzori
Lucinda Grainger
Hana Hassanin MD
James Woolley
Iris Wood-Campar
Janetta Rexha

Helix Centre – Human Centred Design led by Matthew Harrison

Sophie Horrocks
Lenny Naar
Brian Quan

Site Investigators and Key Personnel:

Surrey and Borders Partnership NHS Foundation Trust (Site and Sponsor)

Chief Investigator: Professor Ramin Nilforooshan
Research and Development Managers: Jessica True, Olga Balazikova
Research Co-ordinator: Emily Beal
Clinical Monitoring Team: Chloe Walsh, Matthew Purnell and Nicole Whitethread, Vaiva Zarombaite, Lucy Copps, Olivia Knight, Gaganpreet Bangar, Sumit Dey, Chelsea Mukonda, Jessica Hine, Luke Mallon

Brook Green Medical Centre / Hammersmith and Fulham Site

Principal Investigator: Dr David Wingfield
Research Nurse / Paramedic: Claire Norman
Clinical Studies Officers/Research Technicians: Anesha Patel, Ruby Lyall, Sanara Raza
Research Therapists: Naomi Hassim, Pippa Kirby
LBHF Support: Assistive Technology: John Patterson, Business Development; Mike Law,
Social Services OT: Andy Kenny.
